# Facilitators and Barriers to Elimination of Mother-to-Child Transmission of HIV (eMTCT) at a Tertiary Hospital in Uganda: A Qualitative Study

**DOI:** 10.21203/rs.3.rs-10910337/v1

**Published:** 2026-10-12

**Authors:** Emma Amadriyo, David Christopher Mukasa, Nelson Kalema, Pebalo Francis Pebolo, Joanita Nangendo, Fred C. Semitala, Susan Atuhairwe, Joshua Kitimbo, Mike Kagawa, Kabwigu Samuel, Dan Kaye

## Abstract

**Background:** Despite progress in the elimination of mother-to-child transmission (eMTCT) of HIV, Uganda continues to report substantial numbers of new paediatric HIV infections, frustrating the elimination efforts. Understanding factors that facilitate or hinder eMTCT service delivery and utilization is essential for strengthening program performance. This study explored facilitators and barriers to eMTCT at Masaka Regional Referral Hospital, Uganda **Objective:** This qualitative study explored facilitators and barriers to the elimination of mother-to-child transmission (eMTCT) of HIV among mothers living with HIV and healthcare workers at Masaka Regional Referral Hospital, a tertiary hospital in Uganda, informed by the Capability, Opportunity, Motivation–Behaviour (COM-B) framework. **Results:** Twenty-seven participants (15 mothers and 12 healthcare workers) took part in in-depth interviews, key informant interviews, and two focus group discussions, analyzed thematically. Four themes emerged. Health system facilitators included integrated HIV services and antiretroviral therapy, while barriers included commodity stockouts and staff shortages. Facilitators related to maternal knowledge and behaviour included positive health-seeking practices, while barriers included limited knowledge and delayed HIV testing. Sociocultural facilitators included male partner and peer support, while barriers included stigma and gender-based violence. Structural facilitators included community outreach, while barriers included transport costs, food insecurity, and breastfeeding-related challenges. Strengthening health systems, addressing stigma, and reducing socioeconomic barriers may improve eMTCT outcomes.

